# REAL-TIME HEALTH OUTCOMES AMONG OLDER ADULTS INITIATING MEDICAL CANNABIS FOR CHRONIC PAIN

**DOI:** 10.1093/geroni/igad104.0036

**Published:** 2023-12-21

**Authors:** Yan Wang, Kimberly Sibille, Zhigang Li, Rene Przkora, Seigfried Schmidt, Margaret Lo, Ana Abrantes, Robert Cook

**Affiliations:** University of Florida, Gainesville, Florida, United States; University of Florida, Gainesville, Florida, United States; University of Florida, Gainesville, Florida, United States; University of Florida, Gainesville, Florida, United States; University of Florida, Gainesville, Florida, United States; University of Florida, Gainesville, Florida, United States; Brown University, Providence, Rhode Island, United States; University of Florida, Gainesville, Florida, United States

## Abstract

Background: Rigorous data are needed on the short- and long-term effects of medical cannabis (MC) on older adults with chronic pain. In response, this prospective study combines technology-based ecological momentary assessments (EMA) and in-person visits over 12 months to obtain subjective and objective data on MC’s effects on older adults. Method: The study recruits and follows older adults (50 years or older, 50% male) with chronic pain for one year. Some will have initiated MC (MC group) and others will not use MC (comparison group). Target enrollment will be 328 (1:1 ratio for MC and comparison group) participants. Collection of subjective and objective data occurs at in-person visits (baseline & 12 months) and via smartphone- and sensor-based measurement bursts at 1, 3, 6, and 9 months. The EMA data capture detailed MC use patterns and subjective short-term outcomes (e.g., pain intensity rating). These data will be integrated with objective data collected using a wearable sensor-based Fitbit (e.g., physical activity and sleep). Results: Multilevel modeling will examine changes in real-time symptoms measured by EMA and objective, sensor-based behavioral outcomes. Results will include changes in momentary pain intensity rated on 0-100 visual analog, real-time anxiety and depressive symptoms, daily sleep duration and quality, and level of physical activity. Conclusions: Our results will provide evidence on whether older adults who initiate MC will have greater reductions in real-time pain intensity and more improvements in physical and emotional functioning compared to those in the comparison group.

